# QTrim: a novel tool for the quality trimming of sequence reads generated using the Roche/454 sequencing platform

**DOI:** 10.1186/1471-2105-15-33

**Published:** 2014-01-30

**Authors:** Ram Krishna Shrestha, Baruch Lubinsky, Vijay B Bansode, Mónica BJ Moinz, Grace P McCormack, Simon A Travers

**Affiliations:** 1 South African National Bioinformatics Institute, SA MRC Bioinformatics Unit, University of the Western Cape, Private Bag X17, Bellville 7535, South Africa; 2 Ryan Institute, School of Natural Sciences, National University of Ireland Galway, Galway, Ireland; 3 Retroviruses and Molecular Evolution, CNRS-UPR 9002, Institut de Biologie Moléculaire et Cellulaire, 15 rue René Descartes, Strasbourg 67084, France; 4 School of Life Sciences, University of Warwick, Gibbet Hill Rd, Coventry CV4 7AL, UK

**Keywords:** Quality trimming, Next-generation sequencing, High-throughput sequencing, Phred scores

## Abstract

**Background:**

Many high throughput sequencing (HTS) approaches, such as the Roche/454 platform, produce sequences in which the quality of the sequence (as measured by a Phred-like quality scores) decreases linearly across a sequence read. Undertaking quality trimming of this data is essential to enable confidence in the results of subsequent downstream analysis. Here, we have developed a novel, highly sensitive and accurate approach (QTrim) for the quality trimming of sequence reads generated using the Roche/454 sequencing platform (or any platform with long reads that outputs Phred-like quality scores).

**Results:**

The performance of QTrim was evaluated against all other available quality trimming approaches on both poor and high quality 454 sequence data. In all cases, QTrim appears to perform equally as well as the best other approach (PRINSEQ) with these two methods significantly outperforming all other methods. Further analysis of the trimmed data revealed that the novel trimming approach implemented in QTrim ensures that the prevalence of low quality bases in the resulting trimmed data is substantially lower than PRINSEQ or any of the other approaches tested.

**Conclusions:**

QTrim is a novel, highly sensitive and accurate algorithm for the quality trimming of Roche/454 sequence reads. It is implemented both as an executable program that can be integrated with standalone sequence analysis pipelines and as a web-based application to enable individuals with little or no bioinformatics experience to quality trim their sequence data.

## Background

The advent of pyrosequencing technologies such as the 454 sequencing platform by Roche [1] has revolutionized the field of genomics meaning researchers can undertake large-scale genomic studies that would have been complex, difficult and even impossible prior to such technologies [2-4]. Current 454 technology allows the generation of as many as one million high quality sequence reads with read lengths of up to 1000 base pairs. The production of such large volumes of sequence data means that manual curation of quality and errors, as could be done with traditional Sanger sequencing, is no longer feasible. One of the major limitations of pyrosequencing is that sequence quality is not consistent, either within a read or between reads generated in the same sequencing run [5] and, thus, downstream analysis of such data may be compromised as a result of low quality data [6]. The quality scores for the current generation 454 sequencing platforms are similar to PHRED scores [7] and represent the probability of a base call error at each individually called base in a read [8]. These quality scores range from 0 to 40 and are log-scaled, meaning that scores of 30 and 40 represent a probability of an incorrect base call of 1 in 1000 and 1 in 10000 respectively. As with most sequencing approaches, the quality of sequence data generated using 454 pyrosequencing decreases linearly across a sequence read [5]. Thus, in many instances it is imperative to undertake quality filtering of 454 sequence data prior to subsequent analysis. Quality trimming generally entails some form of iterative removal from one or both ends of a sequence read with the primary goal being to ensure that the resultant read is of high quality. Quality trimming tools range from strict approaches that have zero tolerance of low quality base calls in the output reads through to averaging approaches that maximize read length by allowing the inclusion of a proportion of low quality base calls within an output read [9]. The algorithms used by averaging approaches differ greatly, ranging from approaches such as clean_reads [10] and PRINSEQ [11] that use a window-based approach to iteratively trim sequence reads until the user-defined quality threshold is satisfied within the window, to FASTX [12] that iteratively trims nucleotides from a sequence read until the percentage of low quality bases in a read satisfies a user-defined threshold. While all of the reads in such approaches will satisfy the mean quality score threshold, the algorithms used can result in tools that ‘over-trim’ reads resulting in the loss of data that, if included, would be both high-quality and informative.

Here, we describe a quality trimming algorithm (QTrim) that uses a novel averaging approach that ensures the output of high quality reads from high throughput sequence data while maintaining the maximum possible length of the output sequences. To enable its use by a broad range of researchers, QTrim is available as a standalone executable for individuals with computational expertise and as a web-interface for individuals with little, or no, bioinformatics experience.

## Implementation

QTrim is executed as a standalone software package for command-line use and integration into sequencing analysis pipelines. Two versions of QTrim can be invoked using the python script. The first is a “simple” version that only outputs the trimmed sequence data. A second, “graphical” version (invoked using the –plot option) also outputs graphs plotting the quality score trends across all reads, the prevalence of read lengths and the mean quality scores both before and after trimming. Further, a web-based interface is also available ( http://hiv.sanbi.ac.za/tools/qtrim) for individuals wishing to quality trim their data using the graphical version of QTrim prior to further downstream analysis using other web-based tools.

**Figure 1:**
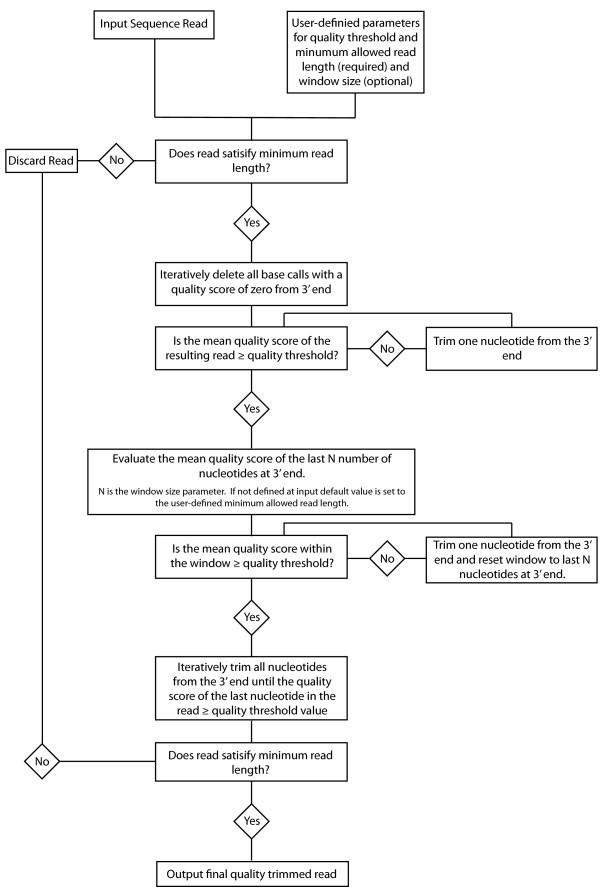
Flow diagram of the various steps involved in the QTrim algorithm.

QTrim takes as input a fastq file or a fasta file with its associated .qual file. QTrim execution requires three parameters to be set by the user. The quality threshold is the mean quality that each trimmed read must satisfy, the second defines the minimum allowed read length (base pairs) a read can reach during trimming before being discarded, while the final parameter (optional) defines the window size to be used during trimming. If no window size is defined at input the default value is set to the user-defined minimum allowed read length. The QTrim algorithm comprises three sequential steps (Figure 1) with the first step iteratively trimming single nucleotides from the 3′ end of a read until it’s mean quality score satisfies the quality threshold. The second step is a sliding window approach that evaluates the mean quality score of the last N number of nucleotides at 3′ end (N is equal to the window size). If the mean quality score of the bases within the window is not satisfied, a single base is deleted from the 3′ end and the window is reset. Once the quality threshold within the window is satisfied, the final step iteratively trims all nucleotides from the 3′ end until the quality score of the last nucleotide in the read ≥ quality threshold value. If the length of the resulting trimmed read is less than the minimum allowed read length the read is discarded. A further option in QTrim enables trimming to occur simultaneously at both the 5′ and 3′ ends.

## Results and discussion

**Figure 2:**
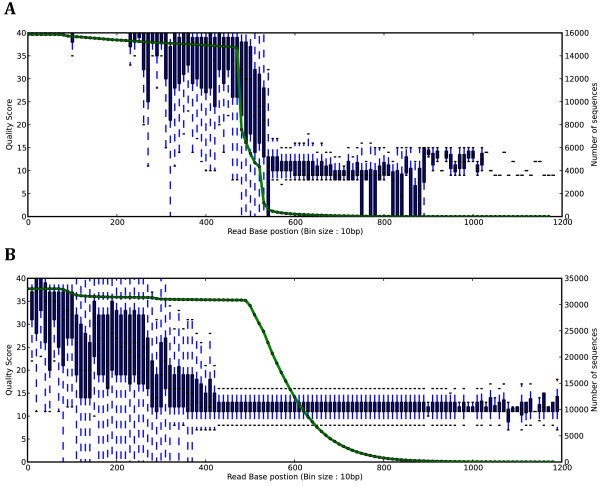
**Box and whisker plots showing an overview of the high quality (A) and low quality (B) data used for comparisons in this study.** The x-axis corresponds to the read base positions (sampled every 10 bp for clarity) while the y-axis corresponds to the quality score. The second y-axis (associated with the green line) corresponds to the read coverage at each of the positions.

The performance of QTrim was evaluated on two previously generated 454 datasets with extreme differences in untrimmed quality scores. While in an ideal world sequencing using high-throughput sequencing platforms would only output high-quality sequence data, the various steps involved in sample preparation and sequencing means that, in many cases, non-optimal sequence data is output from a sequencing run. Rather than discard such data, a sensitive trimming approach will enable individuals to “rescue” any high quality present as the result of a sub-optimal sequencing run. The good quality 454 sequence data used here originates from a metagenomic project sequencing bacterial 16 s genes from seawater sponges (in prep). This data was generated using the Roche/454 FLX platform and good quality was defined as sequence reads with comparable read lengths with consistently high quality scores at called bases until close to 400 bp (Figure 2A). The poor quality input data was also generated using the Roche/454 FLX platform and originated from a study of HIV drug resistance [13]. This data was characterized as having particularly variable read lengths with low quality nucleotide calls prevalent throughout the entire read (as opposed to for all generated sequence reads (Figure 2B).

The performance of QTrim was compared at two quality threshold levels (Q20 and Q30) against PRINSEQ [11], the Lucy algorithm [9] as implemented in clean_reads [10], the modified-Mott algorithm implemented in Geneious [14], FASTX [12] and 454/Roche’s newbler v2.6. Each of the methods was evaluated on the basis of total number of trimmed reads output, the mean read length of the output reads as well as on the basis of trimming speed. For each method, all trimmed reads were confirmed to satisfy the quality threshold (Q20 or Q30) to ensure that there was no bias in the evaluation step towards approaches that favour untrimming to maximize read length by outputting longer reads that do not satisfy the quality threshold. The best approach should output the highest number of trimmed sequencing reads that satisfy the quality threshold with the longest average read length.

**Figure 3:**
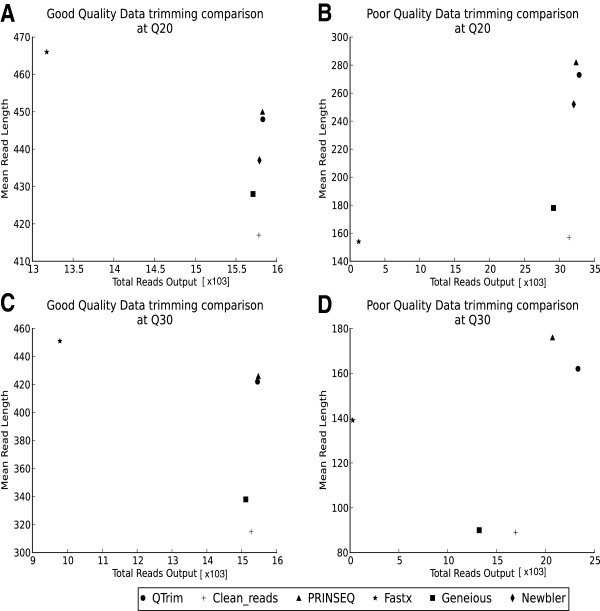
**Comparison of QTrim with other quality trimming approaches on the basis of the total number of reads output versus the mean read length output.** These comparisons were performed using good quality **(A + C)** and poor quality **(B + D)** sequence data at trimming thresholds of 20 and 30.

When applied to the good quality data, QTrim and PRINSEQ perform equally well and outperform all other methods (Figure 3A and C), with 15829 trimmed reads with a mean length of 448 nucleotides output by QTrim and 15825 trimmed reads with a mean length of 450 nucleotides output by PRINSEQ in the Q20 threshold analysis. In the more stringent Q30 analysis, the number of output reads remained similar to that of the Q20 analysis however the mean read length reduced to 422 and 426 nucleotides for QTrim and PRINSEQ respectively. For both the Q20 and Q30 analysis all of the other approaches output a comparable number of trimmed reads to QTrim and PRINSEQ, however their average read lengths were significantly shorter (Figure 3A and C).

When applied to the poor quality data, PRINSEQ and QTrim were, by far, the two best performing approaches (Figure 3B and D) with 32818 trimmed reads with a mean length of 273 nucleotides output by QTrim and 32381 trimmed reads with a mean length of 282 nucleotides output by PRINSEQ in the Q20 threshold analysis. The lower quality of this data is reflected in the much shorter trimmed reads output from the analysis when compared to the trimmed read lengths output by the analysis of the good quality data. This is further evident when the stringent Q30 analysis of the poor quality data was undertaken with the average trimmed read length reducing from 273 nucleotides (Q20) to 162 nucleotides (Q30) for QTrim and from 282 nucleotides (Q20) to 176 nucleotides (Q30) for PRINSEQ. Further, the dramatic reduction in the number of reads output for all methods in the Q30 analysis (ranging from a 29% reduction in the number of high quality reads output between the Q20 and Q30 analysis in QTrim to a 77% reduction in Fastx), indicates that, for many sequence reads, they were of too low quality to pass the minimum read length threshold.

Upon comparison with all other approaches, QTrim performs equally as well as the best of these methods (PRINSEQ). The trimmed reads output by PRINSEQ are, on average, slightly longer than those output by QTrim (Figure 3). Upon further examination, however, this is as a result of PRINSEQ outputting a higher number of low quality bases (quality score < 20) at the 3′ end of its trimmed reads. For example, PRINSEQ output 8% as many low quality bases than QTrim in the Q20 trimming of both datasets tested here, and outputs 17% and 25% as many low quality bases in the Q30 trimming of the poor quality and good quality datasets respectively. We find that this is the case in all of the methods that use an averaging approach for quality trimming. As soon as the minimum quality score in a read satisfies the quality threshold, the read is output as trimmed without any further analysis. In QTrim, however, we employ a further two steps that ensures that low quality bases at the 3′ end of quality trimmed reads are removed. Thus, while the reads may be slightly shorter than those output by PRINSEQ, users can have a high level of confidence that quality is consistent across the length of the quality trimmed reads output by QTrim. Further, as there is no window size that needs to be defined by the user for the initial trimming step, QTrim results are not susceptible to error by a poorly user-defined window size. Setting the window size too small in a windowing approach such as PRINSEQ would mean that while the quality threshold is satisfied within the window, it is not satisfied across the entire output read.

**Figure 4:**
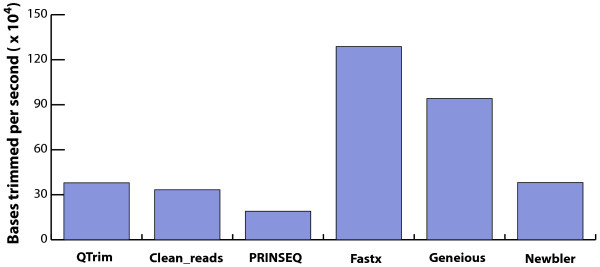
**Comparison of the trimming speeds for all of the methods represented as the number of bases trimmed per second.** Both Geneious and Newbler are graphical software packages and, thus, trimming speeds were estimated based on running time of the entire graphical process as opposed to solely the trimming algorithm.

Finally, the operation of QTrim is twice as fast as PRINSEQ (379372 versus 189966 bases trimmed per second) on a standard desktop computer with a 2 GHz Intel**®** Core**™** Duo CPU and 2GB of RAM Figure 4.

## Conclusion

QTrim is a fast, highly sensitive and accurate algorithm that outperforms all available approaches for quality trimming of 454 sequence data. A noteworthy feature is that it enables sensitive trimming of sub-optimal sequence data thereby enabling researchers to undertake downstream analysis on lesser quality sequence data that otherwise may have been discarded. The command line python version of QTrim can be easily incorporated into sequence analysis pipelines, while the web interface enables users with little or no bioinformatics experience to undertake quality trimming of their high-throughput sequencing data.

## Availability and requirements

**Project name:** QTrim

**Project home page:**http://hiv.sanbi.ac.za/tools/qtrim

**Operating system(s):** MacOS, Linux

**Programming language:** python v2.6 or v2.7 (not v3.0)

**Other requirements:** Executables – Nothing, Source code – Biopython, numpy, Matplotlib

**License:** GNU GPL v3.

**Any restrictions to use by non-academics:** Commercial use may be restricted and such users should contact the corresponding author for further details.

## Competing interests

RKS, BL, VBB, MBJM and GPM have no competing interests. SAT has delivered training workshops in conjunction with Roche (South Africa) as well as having conference attendance sponsored by Roche (South Africa).

## Authors’ contributions

RKS developed the algorithm and implemented the code for the python version of QTrim while BL designed and implemented the QTrim web interface. VBB, MBJM and GPM generated the 454 data used in the study. SAT conceptualized the project, supervised the work and wrote the final version of the manuscript. All authors read and approved the final manuscript.
